# On the rules of life and Kleiber's law: the macroscopic relationship between materials and energy

**DOI:** 10.1016/j.heliyon.2022.e09647

**Published:** 2022-06-06

**Authors:** Benjamin Leiva, John R. Schramski

**Affiliations:** a OES, Universidad Del Valle de Guatemala, Ciudad de Guatemala, Guatemala; b College of Engineering, University of Georgia, Athens, Georgia, USA

**Keywords:** Energy, Mass, Kleiber's law, Metabolic ecology, Limits, Conservation

## Abstract

Efforts to accommodate the growth in global energy consumption within a fragile biosphere are primarily focused on managing the transition towards a low-carbon energy mix. We show evidence that a more fundamental problem exists through a scaling relation, akin to Kleiber's Law, between society's energy consumption and material stocks. Humanity's energy consumption scales at 0.78 of its material stocks, which implies predictable environmental pressure regardless of the energy mix. If true, future global energy scenarios imply vast amounts of materials and corresponding environmental degradation, which have not been adequately acknowledged. Thus, limits to energy consumption are needed regardless of the energy mix to stabilize human intervention in the biosphere.

## 1 Introduction

Humanity's rate of energy consumption (i.e., power) of 16.1 TW in 2010 is projected to increase by 94–247% by 2050, with a reference scenario at 140% (van Ruijven et al., 2019). On track with these expectations, humanity reached 18.9 TW in 2018 - a yearly 2% growth since 2010. Such growth is confronted by the well-documented realities of climate change (Tong et al., 2019), persistent 80% share of fossil fuels in the global energy mix (IEA, 2019), and declining quantity and quality of fossil fuel reserves (Chapman, 2014; Mohr et al., 2015). These issues have highlighted the need for an energy transition towards low-carbon energy sources (Grubler, 2012; Smil, 2016; Sovacool, 2016) such as renewables and nuclear (Moriarty and Honnery, 2016; World Nuclear Association, 2019). The main obstacles discussed in the literature have been slow deployment (Moriarty and Honnery, 2009), low energy return over investment (Hall, 2017; Murphy, 2014; Rye and Jackson, 2018), and the material requirements of new energy technologies, specifically metals such as cobalt and lithium (Giurco et al., 2019; Moreau et al., 2019; Valero et al., 2018).

We show evidence that the continued increase of energy use faces a more fundamental problem. The use of energy requires prime movers such as people, engines, computers (and their supporting infrastructure) that are built from materials found originally in nature. Moreover, the use of energy inevitably rearranges additional materials in the environment. This posits a problem that goes beyond the carbon content and specific material requirements of given technologies, and thus cannot be solved through energy substitution. Higher power rates need and provoke more materials (metals and others) being rearranged from otherwise healthy ecosystems into social structures such as firms, cities, and governments and into goods such as furniture, electronics, and food. In fact, the 20th century witnessed a 9-fold increase in humanity's power alongside a 16-fold increase in its material stocks (Krausmann et al., 2017). We contend that the ecosystem degradation (MEA, 2005), biodiversity loss (IPBES, 2019), and overall human intervention in the Earth system (Steffen et al., 2015) that followed from such harvesting of the biosphere could have only partially been avoided with a low-carbon energy mix (Makarieva et al., 2008a).

The scaling relation between energy use and mass has been well documented in biological organisms. Macroecological theory and Kleiber's Law in particular have shown that in general biological organisms' power is allometrically proportional to its mass with an exponent that tends to 2/3 within species (intraspecific scaling) and 3/4 between species (interspecific scaling) (Enquist et al., 1999; Koziowski and Weiner, 1997; Lovegrove, 2000). Many studies have challenged the adequacy of these rather precise universal values (Dodds et al., 2001; Glazier, 2005; Kozlowski and Konarzewski, 2004; Makarieva et al., 2008b), but there is no doubt that a scaling relation between mass and power exists given the depth and breadth of the literature. Intuitively, living systems use energy in foodstuff through mitochondria, cells, and muscles to rearrange carbon, hydrogen, and other elements to further build and maintain the components of a functional body and then use that body to intervene in the world. The higher the energy use the higher the associated mass and vice versa.

**Figure 1:**
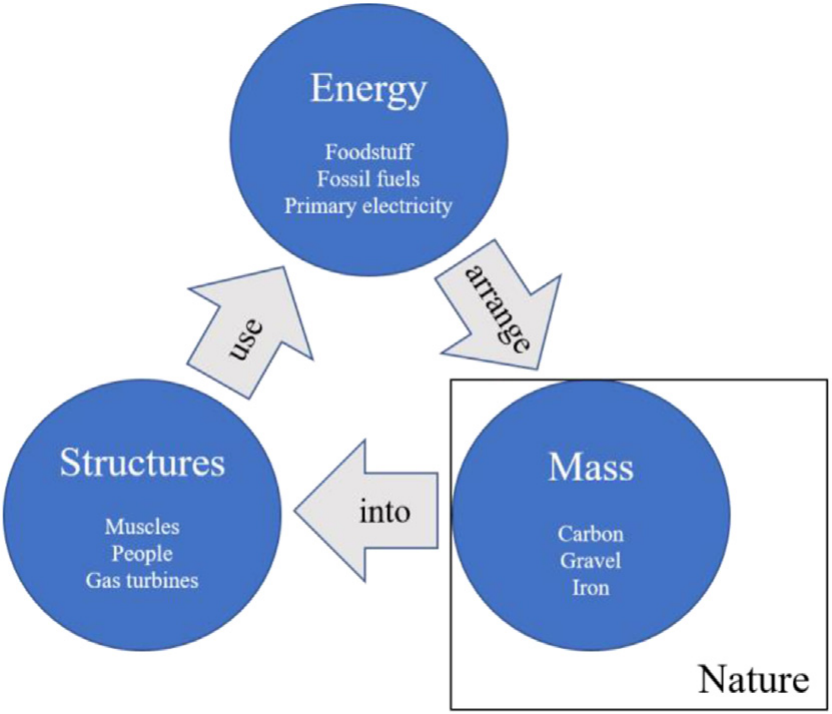
The relation between structures, energy, and mass.

Similarly, social systems act as super-organisms that also use energy to rearrange mass into living support structures, and then use those structures to modify the environment (Figure 1) (Haberl et al., 2019; Krausmann et al., 2017; Rees, 2012). *In a very real sense both animals and economies have “metabolisms”. Both consume, transform, and allocate energy to maintain complex adaptive systems far from thermodynamic equilibrium”* (Brown et al., 2011)*.* The main difference is that social systems use more types of energy sources (e.g., foodstuff, biomass, fossil fuels, electricity), through a broader set of prime movers (e.g., people, gas turbines, computers) to rearrange a wider set of materials (e.g., biomass, gravel, iron, silicon) into the components of a functional society (e.g., people, products, buildings, infrastructure).

We evaluate if a scaling relation akin to Kleiber's Law holds between power and the material stocks of social systems. Previous social allometric scaling studies have related power to birthrate and child mortality (Burger et al., 2011), and power to GDP/capita (Brown et al., 2011), but to the best of our knowledge, this is the first attempt to relate power to mass in social systems as in biological organisms. Material constraints to the energy transition have been pointed out for specific materials (Giurco et al., 2019; Moreau et al., 2019; Valero et al., 2018), but not in a general approach as allowed through allometric scaling. Similarly, input-output models that include physical flows and energy use as in Guevara and Domingos (2017), Liang et al. (2017), and Wiedmann et al. (2007) can show how the growth of specific economic sectors relates to the growth of specific material and energy flows, yet do not focus on the more fundamental relation between them, nor between material *stocks* and energy flows.

This relation implies a strict limit to power growth that cannot be addressed with low-carbon energy sources. Using our results, we show that current projections of population and energy use require unsustainable levels of material stocks from the environment. To be clear, the article does not provide an alternative to model and forecast future energy consumption but uses such forecasts and the relation between energy use and material stocks to predict general material requirements. Given this relation, we show the fundamental tradeoff between population and per capita power at each level of social material stocks. We discuss the implications of the tradeoff in the context of population and power growth projections, current per capita power levels, and notions of “sufficiency” (Steinberger and Roberts, 2010), “saturation” (Martí nez and Ebenhack, 2008), and “thresholds” (Frischknecht, 2007; Schulz et al., 2008).

## 2 Materials and methods

Kleiber's law is conventionally modelled as

(1) $$P∼AM^{\alpha },$$

where P is an organism's power rate typically measured with Basal Metabolic Rate in watts, M is the organism's mass measured in grams, α is the dimensionless scaling relation between both and A is a constant (Koziowski and Weiner, 1997; West and Brown, 2005). A log-transform of both variables in Eq. (1) linearizes the relation and allows estimating α with Ordinary Least Squares (OLS). We used Eicker–Huber–White standard errors to control for heteroscedasticity. Following Warton et al. (2006) we also estimated the model with the major axis (MA) and standardized major axis (SMA) techniques given the importance of the scaling relation estimate ($\hat{\alpha }$) for this study.

We obtained $\hat{\alpha }$ for 1) Humanity, the USA, and Japan independently, 2) jointly alongside biological organisms, and 3) jointly alongside biological organisms but after demeaning each series independently (Figure 3). This last approach eliminates the influence on $\hat{\alpha }$ of the different y-intercepts of the individual series’ best-fit curves.

Using the OLS result for Humanity we estimated the mass associated with future power levels with the inverse of Eq. (1), namely $M=BP^{\beta }$ with β≈1/α. We selected the OLS method as this is best for predictions (Warton et al., 2006). The confidence interval on the additional material stocks was estimated with the lower bound of the conservative future power prediction and the upper bound of the generous future power prediction. This same specification was used to forecast the mass associated with different population levels at 2.0 kW/capita and with current per capita power projections. This is the basis of the three-dimensional graphs depicting the relation between a continuum of materials, population, and per capita power levels (Figure 5).

The data was compiled from published estimates of power and material stocks of Human civilization between 1900-2010 and the USA and Japan between 1980 and 2005 (Table 1). Humanity's material stocks come from Krausmann et al. (2017) and those of the USA and Japan from Fishman et al. (2014). Both sources contain the same non-human types of mass. Human mass, considered as population times average weight, was inconsequential.

**Table 1:** Descriptive Statistics for mass, power and their log transforms.

Variable	Definition	USA (n= 26)	Japan (n= 26)	World (n= 111)
		Mean (Range)	Mean (Range)	Mean (Range)
*P*	Power rate in watts	2.7e+12	5.9e+11	6.0e+12
		(2.2e+12–3.2e+12)	(4.4e+11 - 6.9e+11)	(1.4e+12 - 1.6e+13)
*Ln(P)*	Natural log of power	28.63	27.10	29.13
		(28.44–28.79)	(26.82–27.26)	(27.95–30.41)
*M*	Mass stocks in grams	8.3e+16	2.9e+16	2.1e+17
		(6.2e+16 - 1.1e+17)	(1.7e+16 - 3.9e+16)	(3.5e+16 - 7.9e+17)
*Ln(M)*	Natural log of mass stocks	38.94	37.86	39.38
		38.67 - 39.21	37.38–38.20	38.10–41.21

Humanity's power comes from Smil (2017) and BP (2019). Data between 1900 and 1910 was linearly extrapolated. Power for the USA and Japan comes from U.S. Energy Information Administration (2019). Mass and power for biological organisms comes from Makarieva et al., 2008b for prokaryotes, protozoa, aquatic invertebrates, amphibians, fish, reptiles and birds. We used the mass-specific power at 25 degrees Celsius and multiplied it by each species' mass to obtain power rates in Watts. Data for mammals was obtained from Ramsey and Schafer (2013) as such taxa was not publicly available in Makarieva et al., 2008b.

**Figure 2:**
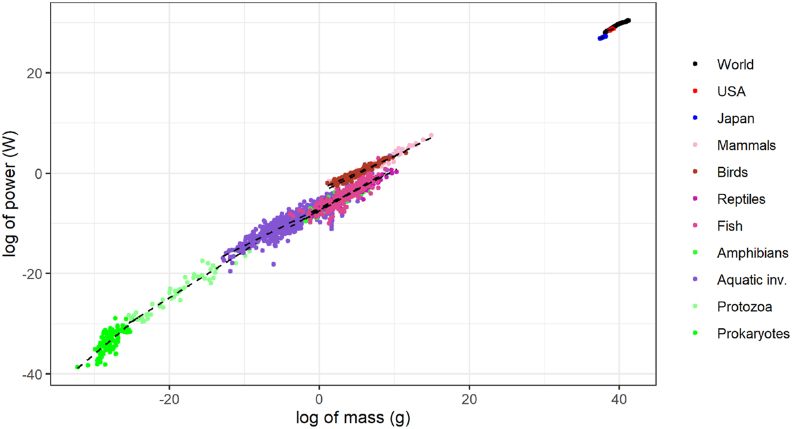
The relation between material stocks and energy for different taxa of biological organisms and social systems (n=2539). Lines are the Ordinary Least Squares (OLS) best fit curves estimated for each taxon and social system independently. The slope of these curves tends to fall as the mass of taxon increases.

Data and code are available as supplementary information. Data on power and mass for social systems and biological organisms is available in the file “Data”, and the code to obtain regression results and Figure 2 through Figure 4 in the file “GeneralCode”. Data to obtain Figure 5 is available in the file “Data3Dmap” and the code to obtain that figure is in the file “Code3Dmap”.

## 3 Results

Our data shows that power and material stocks in the USA, Japan, and for Humanity seem to be related in a manner consistent with Kleiber's Law regardless of the specific modelling technique (Table 2). The exponents for the USA and Japan are not statistically different from 2/3, which is the typical value for intraspecific scaling and thus coincidental with the idea of an individual country developing over time. The exponent for Humanity as a whole is not statistically different from 3/4, which is the typical value for interspecific scaling and thus coincidental with the developing path of many different countries.

**Table 2:** Scaling parameter estimate ($\hat{\alpha }$) - Social systems.

System	OLS		MA		SMA	
	$\hat{\alpha }$	95 % CI	$\hat{\alpha }$	95 % CI	$\hat{\alpha }$	95 % CI
*USA (n = 26)*	0.665	0.601–0.730	0.676	0.613–0.744	0.683	0.621–0.750
*Japan (n = 26)*	0.611	0.548–0.674	0.621	0.558–0.687	0.629	0.568–0.695
*World (n = 111)*	0.783	0.765–0.801	0.788	0.770–0.806	0.789	0.771–0.807

Combining these three super-organisms with a sample of biological ones relates power and mass over 29 and 31 orders of magnitude respectively which represents the widest range studied in the literature. The resulting exponent is significantly higher than 3/4 (Table 3), which is partly due to the different y-intercepts of the individual series’ best-fit curves. Independently centering each series to eliminate their y-intercepts and then regressing them together yields an exponent not statistically different from 3/4 with OLS, yet significantly higher with MA and SMA (Table 3).

**Table 3:** Scaling parameter estimate ($\hat{\alpha }$) - Combined.

System	OLS		MA		SMA	
	$\hat{\alpha }$	95 % CI	$\hat{\alpha }$	95 % CI	$\hat{\alpha }$	95 % CI
*Combined (n = 2539)*	0.914	0.909–0.918	0.921	0.916–0.925	0.921	0.917–0.926
*Demeaned (n = 2539)*	0.762	0.750–0.775	0.815	0.802–0.829	0.829	0.816–0.841

Note: 163 observations from social systems and 2,376 from biological organisms.

These results tend to reject universal 3/4 scaling, which is not surprising given that the majority of the biological data comes from Makarieva et al., 2008b where *“results exclude the possibility of such a universal dependence”.* In fact, graphing these data together with distinct OLS best-fit curves shows a tendency of decreasing slopes at higher massed taxa, specially at the smallest scales (Figure 2).

**Figure 3:**
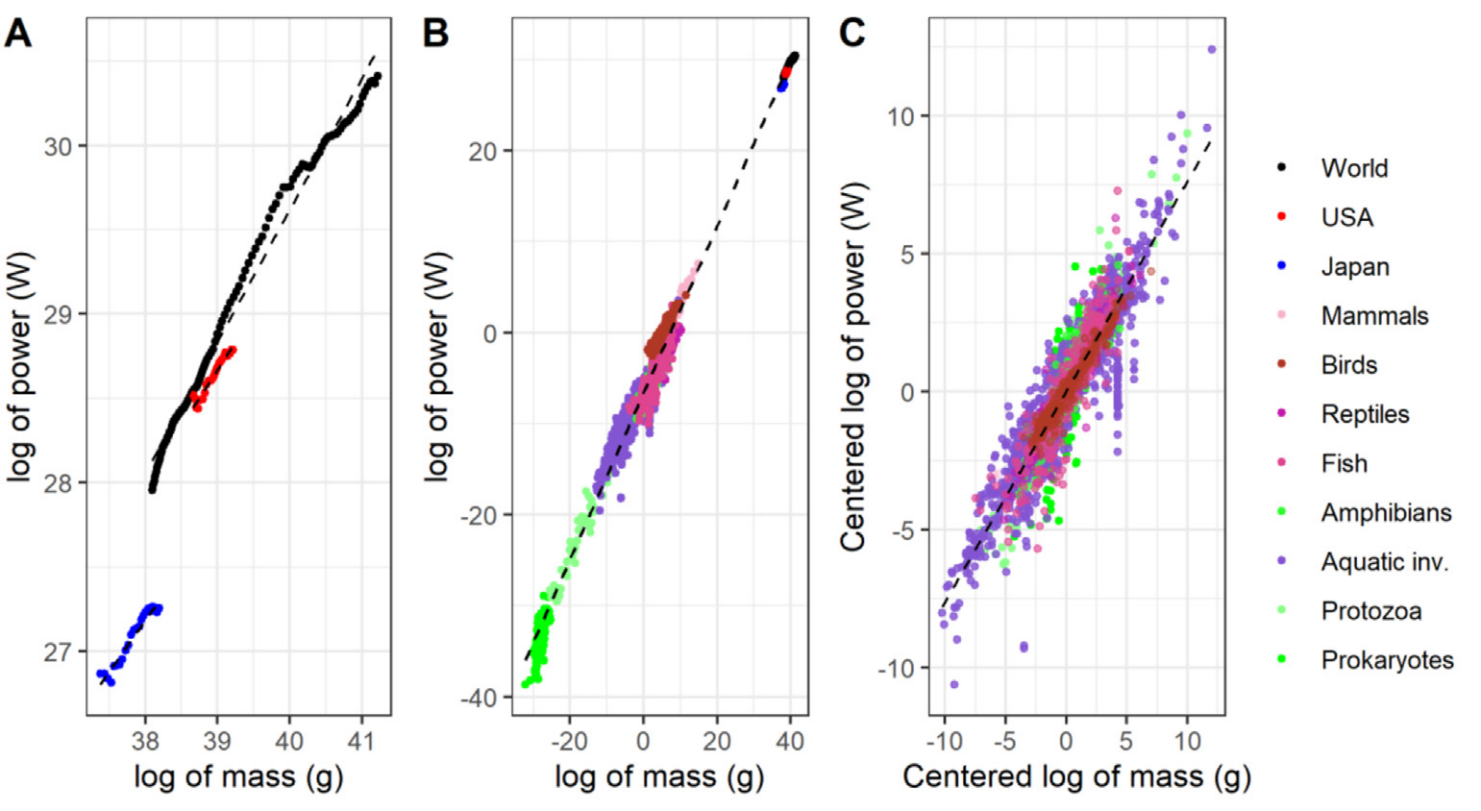
The relation between material stocks and energy. Panel A shows the scatter plot for Japan in blue (n=26), the USA in red (n=26), and globally in black (n=111). Lines are the individual Ordinary Least Squares (OLS) best fit curve. Panel B depicts the information from Panel A in the top right with a sample of biological ones from different taxa in the bottom left (n=2376). The line is the OLS best fit curve for all data (n=2539). Panel C shows the same scatter plot but demeaning each social system and taxa independently. The line is the OLS best fit curve for all the demeaned data. Data is referenced and explained in Materials and Methods.

Yet these are second-order issues given our inquiry is the very existence of a scaling relation *akin* to Kleiber's Law at social scales. The evidence at hand indicates that in social systems there is a scaling relation between mass and energy. Graphing the data for the social systems with their OLS lines fitted independently shows the tight path that these super-organisms have followed while growing (Figure 3 – Panel A). Moreover, plotting that data alongside the one for biological organisms and a unique OLS best-fit line shows how complex systems, ranging from prokaryotes, protozoa, through reptiles and mammals up to human civilization, scale their mass and energy by roughly 30 orders of magnitude with relatively little deviation (the OLS best-fit curve yields an R^2^ of 98.44) (Figure 3 – Panel B). In fact, eliminating the scale differences by demeaning each series independently yields a dense cloud of points that disguises the social system's data and results in an OLS best-fit curve with an R^2^ of 84.77 (Figure 3 – Panel C).

**Figure 4:**
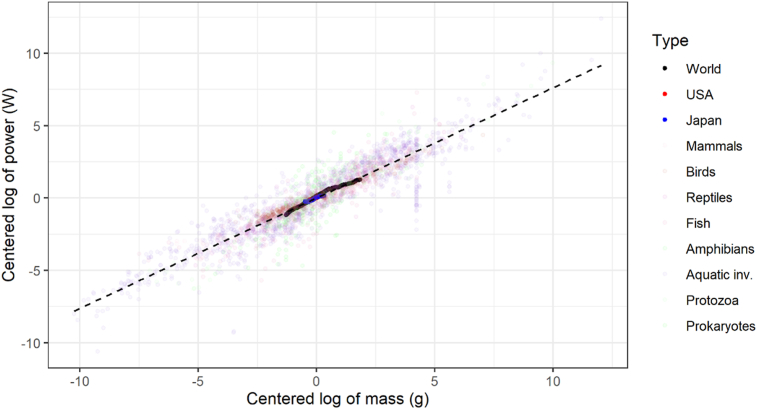
Panel C of Figure 3 with transparency of biological data.

A modified version of Panel C with biological data made transparent helps see the behavior of social systems' data when demeaning (Figure 4). Social systems show a tight trajectory over the combined OLS line such that their data lies on top of each other.

## 4 Discussion

Why do social systems' power and mass scale this way? One idea is based on fractal geometry (West et al., 1999), where the invariant “length” could be the individual person. Brown et al. (2011) use this idea arguing that “*The energy and other resources that sustain these systems [animals and economies] are supplied by hierarchically branching networks, such as the blood vessels and lungs of mammals and the oil pipelines, power grids, and transportation networks of nations. Models of these networks suggest that three-quarter-power scaling optimizes distribution of resources*”.

Another idea is based on combustion science and oxygen (O_2_) concentrations. Efficiency of thermal transfer is dependent on oxygen concentrations which control combustion rates and the rates of ATP production and destruction in biological systems. Contrasting combustion and metabolism have further defined the relationship between mass and energy in energy transactions. For example, in biology low O_2_ concentrations can trigger a step-function drop in ATP concentrations which subsequently promotes a harsh shift towards a much less efficient glycolysis pathway. Boundaries like these are explored with sophisticated oxygen deficient metabolism models using a “cloud” of cells that are together functioning as an organ or models using a “cloud” of organs that are together functioning as an organism. These models have also shown Kleiber like scaling coefficients as apparent outcomes of multi-scaled self-organization and these outcomes have applicability to the expected carbon clouds, O_2_ concentrations, and thermal efficiency of combustion theory (Annamalai, 2021).

Other possible interpretations could be based on size-dependent limitations of resource storage, where the role of macromolecules would be played by energy goods (Maino et al., 2014; Thommen et al., 2019), and the interaction of physiological features with environmental conditions, where growth and reproduction could be given by an economy's aggregate investment and consumption (Koziowski and Weiner, 1997).

In any case, noting that the theoretical basis of Kleiber's Law remains controversial after 80 years of research (Escala, 2019; Hulbert, 2014), finding a theoretical explanation for the apparent validity of Kleiber's Law at social scales exceeds the scope of this paper and remains as future research that may become foundational science towards Humankind's sustainability. An important caveat in the analogy is that allometric scaling is studied with resting energy throughput (i.e., Basal Metabolic Rates (BMR)), whereas this study used total energy used by societies. Subtracting energy used for growth to have a closer measure of “social BMR” and obtaining further data on mass and energy of nations is important future research to broaden the empirical basis of this relationship. Data from sufficient countries could show variability from the 2/3 exponent found here for the USA and Japan, perhaps due to differences in environmental temperature, development strategies, economic structure, or cultural traits.

The main implication of these findings is that, regardless of the energy mix, future energy growth scenarios are fundamentally proportional to the rearrangement of prodigious amounts of materials. Given $P∼M^{0.78}$, for a projected population of 9.7 billion and per capita power consumption of 4 kW/capita, the resulting global power growth of 140% between 2010 and 2050 will result in Humanity's material stocks increasing by 1109 Gt (95% CI 959–1271 Gt). The actual amount of raw materials taken from nature would be higher because more than one ton of materials must be extracted from nature per ton of materials included in civilization (Krausmann et al., 2018). Given that Humanity's current material stock is roughly 800 Gt, how can an increase to 1909 Gt take place while maintaining the biosphere's integrity? Estimates of Earth's green matter, for example, vary from 268 to 901 Gt C with 500–800 Gt C being the generally accepted range (Smil, 2013).

**Figure 5:**
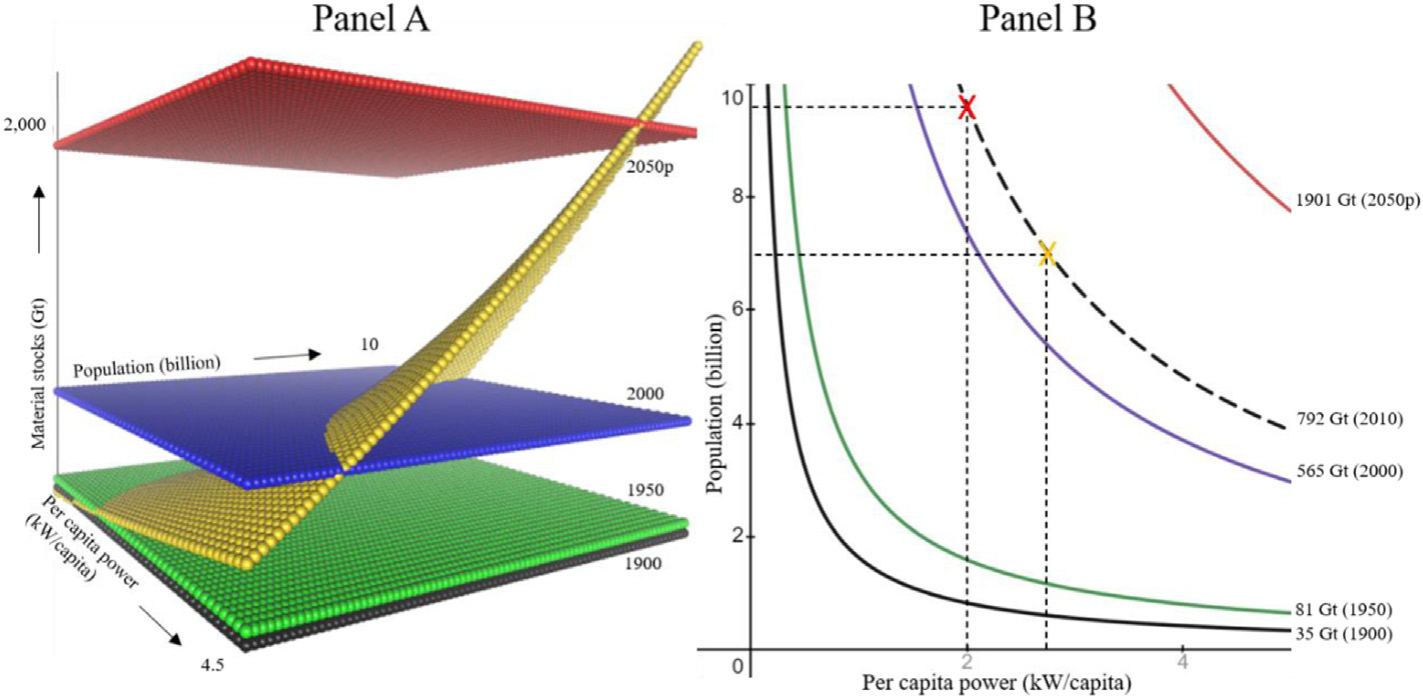
Panel A depicts the relation between per capita power, population, and material stocks. The yellow plane shows the 0.78 scaling of material stocks as a function of per capita power and population. In 1900, a population of 1.6 billion people averaging 0.8 kW/capita implied material stocks of 35 Gt (black plane). In 1950, a population of 2.5 billion averaging 1.3 kW/capita implied materials stocks of 81 Gt (green plane). In 2000, a population of 6.1 billion people averaging 2.1 kW/capita implied material stocks of 565 Gt (blue plane). In 2050, a projected 9.7 billion people averaging 4.0 kW/capita implies material stocks of 1901 Gt (red plane). Note the rapidly increasing distance between the material stock planes at equal 50-year intervals. Panel B shows the intersections of the yellow plane at each material stock plane of Panel A to depict iso-material stock curves. Iso-material stock curves are the compensation between population and per-capita power to maintain a given level of material stock levels. These curves only show point estimates of the mathematical scaling relation and therefore need not show the exact historical values of population and per capita power. The dashed-black line in Panel B shows the 2010 iso-material stock curve (2010 level not shown in Panel A). The line depicts 792 Gt of material stocks with 6.9 billion people averaging 2.8 kW/capita (orange marker, actual data for 2010 is 6.9 billion people averaging 2.3 kW/capita). Data is referenced and explained in Materials and Methods.

Trivially, the relation found here implies that material stocks simultaneously scale with per capita power and population growth (yellow plane, Figure 5 – Panel A). The levels of growth in material stocks are shown in 50-year intervals (black, gray, blue, and red planes). The level of material stocks in 1950 (gray) coincide with the onset of the Great Acceleration. The increasing distances between the material-stock planes at 50-year durations depicts the changing speed in material use of the Great Acceleration.

The maximum sustainable level of material stock is currently unknown and undefined. It is likely that 1901 Gt (projected 2050 level) is unsustainable and dangerous, while 81 Gt (1950 level, just before the Great Acceleration) is likely sustainable and safe. The 2010 level of 792 Gt might already be unsustainable, considering that by then humanity had trespassed at least two planetary boundaries (Steffen et al., 2015), yet it could be sustainable if ecosystem protection, greener agriculture and low-carbon energy sources were implemented. In any case, setting that year's material stocks as the maximum sustainable level helps begin the discussion of tradeoffs between per capita power and population. If society's power and material stocks scale as suggested, the balance of these tradeoffs is foundational for sustainability science. For example, a goal to maintain material stocks in 2050 at the 2010 level (792 Gt) with a projected population of 9.7 billion would require no more than 2.0 kW/capita (Figure 5 – Panel B, dotted line, red marker).

These results include technological progress and thus the tradeoffs cannot be avoided through it. For example, during the 20th century Humanity's power scaled at α= 0.78 and not proportionally to mass at α= 1 because, for example, the energy cost of ammonia dropped from over 100 to 33 MJ/kg (Smil, 2001), of iron from over 50 to 10 MJ/kg (Smil, 2008), of aluminum from 50 to 13 MJ/kg (Smil, 2005), light bulbs' efficiency improved from less than 25 to more than 175 lumen/W (Smil, 2003), and engines reduced their mass-to-power ratios from 90 to less than 1 g/W (Smil, 2005). Without these and other technological achievements power would have scaled proportionally to mass, and with α= 1 humanity's 2010 material stock level would have been associated with power 8,000 times higher. If social systems obey a scaling relation as suggested, this relation between power and mass becomes a constraint for social metabolism as fundamental as in animal metabolism (West and Brown, 2005), and technological innovation plays a role in the former as important as evolution has played in the latter.

Absent technological fixes (and population reductions), the most reasonable response to ecological overshoot and the relation between social mass and power would be a worldwide 2.0 kW/capita limit. The implementation of such limit poses gargantuan technical and political challenges. Given the difficulty and invasiveness of individual measurements of energy consumption, how could a country enforce it? And thus, how could a community of countries do so? Moreover, even if technically possible, it is unlikely that any country would willingly reduce its average per capita power (Smil, 2008). Historically this has only happened under extreme circumstances such as the fall of the Roman Empire and the breakdown of the Soviet Union. A peaceful and ordered lowering to 2.0 kW/capita would be unprecedented in medium-high-powered countries with 3.0 kW/capita (e.g., China, Chile), let alone in very-high-powered countries with more than 10.0 kW/capita (e.g., USA, Australia, Canada). In all, 53% of the 151 countries with data in 2014 had more than 2.0 kW/capita. In 2019 such percentage rises to 78% between the 80 countries with data, due to the growth in per capita power and selection bias among countries with the most updated information.

Although unprecedented, we contend that such reduction of per capita power is needed regardless of the energy mix given the relation of mass and energy found here. Failing to do so risks disrupting the biophysical foundations of human civilization, and triggering waves of civil unrest and violent conflict in the process (Ahmed, 2017). Fortunately, there is extensive literature documenting asymptotic social returns from increasing power for the Human Development Index (HDI) (Annila and Kuismanen, 2009; Arto et al., 2016; Lambert et al., 2014; Martí nez and Ebenhack, 2008) and other important social indicators such as political freedom and improved water access (Arto et al., 2016; Goldemberg et al., 1985; Pasten and Santamarina, 2012; Schulz et al., 2008; Smil, 2008). Intuitively, at low levels of per capita power (e.g., <1.0 kW/capita) more power helps secure the necessities of life, but over a certain threshold (e.g., >3.0 kW/capita) such necessities have already been satisfied and therefore more power does not lead to better social outcomes. Historically, early hunter-gatherers used just enough energy to satisfy their metabolic needs of ∼0.1 kW/capita. Power per capita increased throughout the agricultural and successive industrial revolutions such that the current global average is ∼2.3 kW/capita and surpasses 10 kW/capita in some high-income countries. A specific limit at 2.0 kW/capita has been proposed by the 2000-watt society since 1998 based on the per capita power of western Europe during the 60s and the dignified life it enabled (Frischknecht, 2007; Schulz et al., 2008). Coincidentally, according to our results 2.0 kW/capita is the value required to maintain material stocks in check below 800 Gt globally by 2050 given the projected population levels.

Limits to per capita power does not necessarily imply limits to consumption, at least in the short run. Radical increases in resource productivity as outlined in Grubler et al. (2018) and circular economy proposals (e.g., Ellen MacArthur Foundation (2013)) could enable some growth in per capita consumption without more energy use and material stocks, or their reduction without sacrificing living standards. This idea is highlighted in Krausmann et al. (2017) when calling for the decoupling between material stocks and the services they provide.

In any case, future efficiency improvements must be accompanied by strict limits to aggregate energy use and corresponding material stocks to avoid the rebound effect (e.g., Jevons paradox) that has prevented previous efficiency gains to translate into lower environmental pressure. Another approach would be to directly reduce working time and per capita consumption of good and services, which could be welfare-enhancing in high-powered societies (Pullinger, 2014) but would require a profound paradigm shift (Brand-Correa and Steinberger, 2017; Daly, 2014; Kuhn, 1962; Schmelzer, 2015; Vanhulst and Beling, 2014). Perhaps the goal of a simpler yet wholesome life is more critical than the energy transition for Humankind's sustainability.

## Declarations

### Author contribution statement

Benjamin Leiva: Conceived and designed the experiments; Performed the experiments; Analyzed and interpreted the data; Contributed reagents, materials, analysis tools or data; Wrote the paper.

John Schramski: Analyzed and interpreted the data; Contributed reagents, materials, analysis tools or data; Wrote the paper.

### Funding statement

Benjamin Leiva was supported by CONICYT PFCHA/DOCTORADO BECAS CHILE/2015 - 72160256.

### Data availability statement

Data included in article/supplementary material/referenced in article.

### Declaration of interests statement

The authors declare no conflict of interest.

### Additional information

No additional information is available for this paper.

## Appendix A Supplementary data

GeneralCode GeneralCode

Data3Dmap Data3Dmap

Data Data

Code3Dmap Code3Dmap
